# Assessing the Impact of a Service Excellence Program on Improving Patient Experience at Primary Health Care Centers

**DOI:** 10.7759/cureus.43749

**Published:** 2023-08-19

**Authors:** Abdullah S Alkahtani, Ammar H Abbas, Abdulaziz M Bin Rsheed, Abood F Alabood, Abdulaziz A Alqahtani

**Affiliations:** 1 Department of Preventive Medicine, Prince Sultan Military Medical City, Riyadh, SAU; 2 Department of Family and Community Medicine, Prince Sultan Military Medical City, Riyadh, SAU

**Keywords:** healthcare quality training, patient satisfaction, patient experience, quality education, primary care centers, primary care, quality improvement, care excellence

## Abstract

Background: Frontline clinical staff play a crucial role in shaping patient experience and, therefore, require adequate training and education to deliver exceptional service. This study aimed to assess the impact of a service excellence and quality training program on frontline clinical staff's knowledge, perception, and attitude toward patient experience and service excellence. We also examined the effect of this intervention on patient experience scores.

Methods: This study utilized a pre-post intervention design, where frontline clinical staff completed a questionnaire to establish baseline "knowledge and perception scores" and "attitude scores" related to patient experience. The same questionnaire was administered after the training program to measure any changes in scores. Patient experience scores were collected from existing data, comparing results from different quarters before and after the intervention. The training and education program covered various components, including effective communication, empathy, service standards, and service recovery.

Results: A total of 256 staff members, including nurses, physicians, and allied healthcare professionals, participated in the training program. The study found statistically significant improvements in all components of staff knowledge, perception, and attitude. Specifically, there was a median improvement in knowledge and perception scores, which increased from 77.2% to 96.5%. Additionally, attitude scores showed a median improvement from 73.8% to 92.5%. Moreover, the overall patient experience score increased from 62% to 72.4%.

Conclusion: The study revealed significant improvements in staff knowledge, perception, and attitude, along with enhanced patient experience scores. These findings suggest a potential role for targeted interventions in enhancing patient experience and supporting ongoing quality improvement in primary healthcare settings.

## Introduction

The top priority for any healthcare system is achieving clinical excellence. This requires continuous efforts in high-quality training, regular peer review, and effective clinical audits, all geared toward ensuring positive patient outcomes and improved health [1,2].

However, clinical excellence can be overshadowed from the patient's viewpoint due to a subpar overall experience. Long waiting times, inadequate privacy, impersonal care, and ineffective communication between healthcare professionals and patients can tarnish the perception of care quality [1,2]. 

Patient experience is a critical aspect of medical care, encompassing patients' actual experiences and feedback. It plays a vital role in assessing care quality, with policymakers worldwide increasingly interested in collecting patient experience data to evaluate providers using different performance indicators and improve healthcare quality [3-5].

The available research literature shows mixed findings on the association between patient experiences and clinical outcomes. Some studies suggest that improved patient experiences are linked to better clinical outcomes like lower rates of complications and mortality, while others do not report such a connection [6-13]. Nonetheless, patient experience remains an important outcome on its own, significantly impacting overall healthcare quality [13].

Despite the growing interest in patient experience, there remains a need to explore the specific interventions that can enhance the experience and their potential impact on healthcare providers [14]. In light of this, our study aims to assess the impact of a comprehensive training and education program on frontline clinical staff's knowledge, perception, and attitude toward patient experience and service excellence. Furthermore, we seek to investigate the effect of this intervention on patient experience scores.

Frontline clinical staff play a crucial role in shaping the patient experience, making them the target of our intervention. The training and education program aims to equip these staff members with the necessary skills and knowledge to deliver exceptional service and patient-centered care. Prior research has indicated that targeted training programs for healthcare professionals can positively influence patient experiences, further reinforcing the significance of our study [14].

The training and education program spans various topics, including effective communication, empathy, patient-centered care, and conflict resolution. By focusing on these essential aspects of patient care, we aimed to empower frontline clinical staff to create a more positive and satisfactory patient experience.

## Materials and methods

 Study design and study population

This study employed a pre-post intervention design to assess the impact of a training and education program on frontline clinical staff in the primary care setting. The participants, including nurses, physicians, and allied healthcare professionals, completed a questionnaire before the intervention to establish baseline "knowledge and perception scores" and "attitude scores" related to patient experience. The same questionnaire was administered after the training program to evaluate any changes in scores.

The study was conducted at two primary health care (PHC) centers, Alwazarat and Alsalam, which operate under the patronage of Prince Sultan Military Medical City in Riyadh, Saudi Arabia. A total of 317 staff members were trained as part of the intervention. Among them, 256 staff members were directly involved in patient care and participated in the analysis. The remaining 61 staff members, although trained, were excluded from the final analysis due to their lack of direct clinical duties. This exclusion allowed us to focus on assessing the impact of the training on frontline clinical staff who have direct interactions with patients.

The intervention

The intervention utilized in this study was a comprehensive training program led by a well-trained instructor and conducted in a four-hour in-person session. The course material covered various essential topics, including service standards, service recovery, effective communication, empathy, patient-centered care, and conflict resolution. The training program was partially adapted and modified from the works of Kennedy et al., ensuring its alignment with established best practices in service quality education [14-16].

The program's core content encompassed crucial aspects such as understanding patient expectations and perceptions, value creation, and the science of service quality. Participants were introduced to various tools and frameworks for measuring and improving service quality, including the service quality gaps framework and strategies for achieving customer delight. For further details on the specific content and curriculum, interested readers may refer to the original works by Kennedy et al. [14-16].

Data collection

Data collection for the clinical staff involved the administration of a baseline and post-intervention questionnaire, which comprised three sections. The questionnaire was developed by a group of experts specializing in patient experience and was made available in both Arabic and English languages to ensure inclusivity and ease of response for the participants.

Prior to the main data collection, the questionnaire underwent a pilot phase, where it was administered to a sample of 19 clinical staff members. The pilot phase aimed to evaluate the clarity, relevance, and appropriateness of the questionnaire's items. Based on the pilot feedback, minor adjustments were made to enhance the questionnaire's overall effectiveness.

In Section A, demographic and professional data of the participants were collected. Section B consisted of 13 questions related to the participant's knowledge and perception of patient experience in PHCs, all of which were measured using a Likert scale ranging from 1 to 5, except for two questions that had a correct answer. In Section C, data were gathered on attitudes and practices that may influence patient experience in PHC settings. Section C comprised eight questions, which were also assessed using a Likert scale ranging from 1 to 5.

Patient satisfaction measurements were collected from an existing patient experience measurement system operated by Press Ganey Associates [17]. The system utilized a modified version of the Hospital Consumer Assessment of Healthcare Providers and Systems (HCAHPS) survey to assess and capture feedback from patients regarding their healthcare experiences. Further details on the content, methodology, and validation of this instrument can be found elsewhere [18].

Two metrics were obtained from the system. Firstly, the system captured and summarized patient comments, which were classified into four categories: positive, improvement opportunity, mixed, and neutral. Secondly, the system assessed the overall performance of the facilities, reporting data for various domains such as access, moving through the visit, nursing and assistant staff, care providers, personal issues, and overall assessment. This assessment allowed tracking the patient's experience throughout their visit to the PHC facility. The data reported by the second system were compared against a preset target for each domain during the quarterly evaluation period. Metrics from three quarters (Q) were compared: Q2 of 2022 served as the baseline, Q1 2023 marked the time of intervention implementation, and Q2 2023 was used for the post-intervention comparison.

Statistical analysis

The knowledge and perception scores were calculated based on a questionnaire comprising 13 questions. Among these, 11 questions were scored on a scale of 1 to 5, and two questions received either 1 point for a correct response or 0 points for an incorrect response. The total scores for all questions were summed up for each participant, with a maximum achievable score of 57. The raw scores were then converted to percentages.

Likewise, the attitude scores were derived from an eight-question section, where all questions were scored on a scale of 1 to 5. The total scores for all questions were summed up, resulting in a maximum attainable score of 40. The raw scores were subsequently converted to percentages.

The knowledge and perception scores, as well as the attitude scores, were not normally distributed. Descriptive statistics, including median, range, counts, and frequencies, were used to summarize the data. The Wilcoxon signed-rank test was applied to compare baseline scores with post-intervention scores, given the non-normal distribution. A significance level of 0.05 was used for all tests. Data analysis was performed using IBM SPSS Statistics for Windows, Version 24 (Released 2016; IBM Corp., Armonk, New York, United States).

## Results

In the study, 256 participants were analyzed, showing a nearly equal gender distribution of 53.5% female and 46.5% male. Among the participants, 50% held a bachelor's degree, with nurses comprising the largest professional group (45.7%), followed by physicians (30.1%) and allied health professionals (24.2%). Additionally, 46.9% of participants had 3 to 10 years of experience in PHC, while 39.8% had received prior training in enhancing patient experience. Table 1 presents the characteristics of the participants.

**Table 1:** Characteristics of the participants (N= 256). PHC: Primary health care

Characteristic	Frequency (%)
Gender	
Male	119 (46.5)
Female	137 (53.5)
Highest educational level	
High school	14 (5.5)
Diploma	39 (15.2)
Bachelor's degree	128 (50)
Master's degree and above	75 (29.3)
Professional role	
Nurse	117 (45.7)
Physician	77 (30.1)
Allied health professional	62 (24.2)
Length of PHC experience	
< 3 years	25 (9.8)
3 – 10 years	120 (46.9)
11 – 20 years	90 (35.2)
> 20 years	21 (8.2)
Previously trained in enhancing patient experience?	
Yes	102 (39.8)
No	154 (60.2)

**Figure 1:**
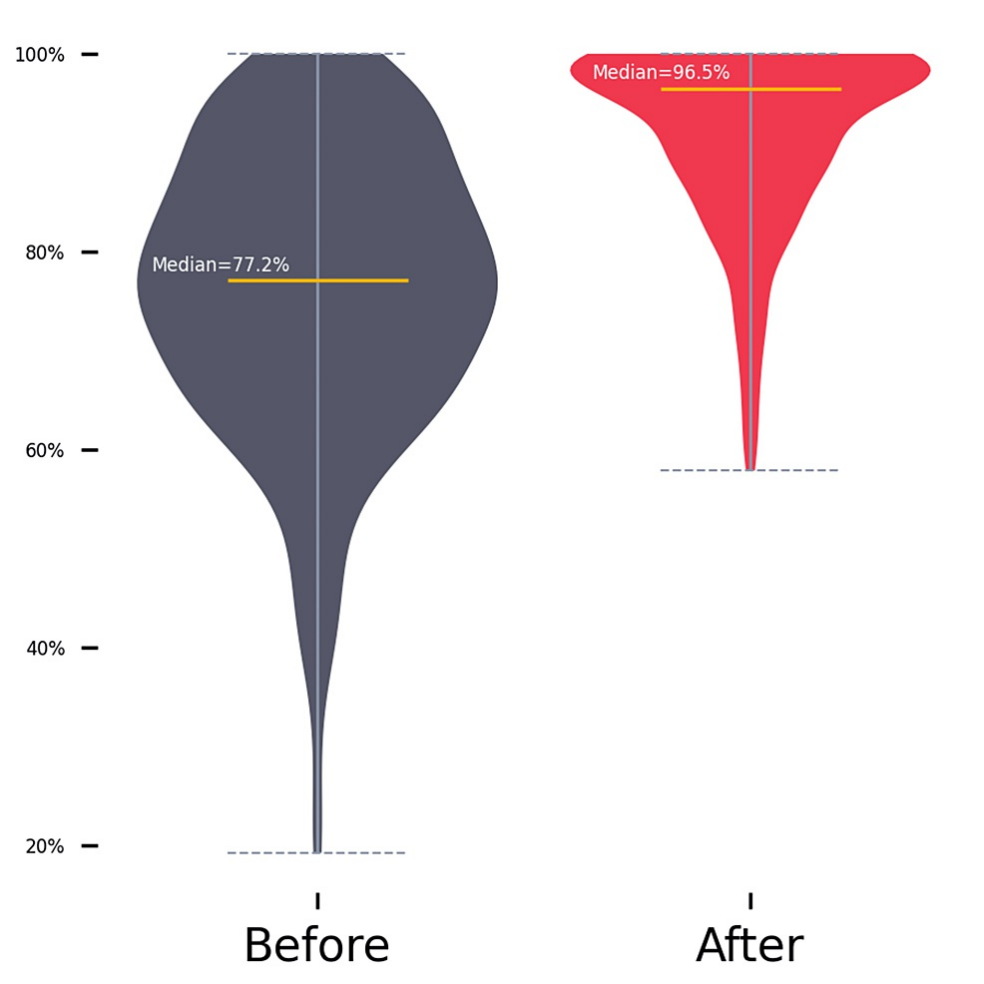
Distribution of pre-intervention (before) and post-intervention (after) knowledge and perception scores.

Figure 1 shows the distribution of pre-intervention and post-intervention knowledge and perception scores using a violin plot.

There was a statistically significant improvement in knowledge and perception scores (Z = 12.599, p < 0.001). The median post-intervention scores (median = 96.5) were significantly higher than the median pre-intervention scores (median = 72.2), with a large effect size (r = .78). Subgroup analysis for knowledge and perception scores is detailed in Table 2.

**Table 2:** Subgroup analysis for knowledge and perception scores. ^*^:^ ^Percentages; ^†^: Wilcoxon signed-rank test; PHC: primary health care

Characteristics	Pre-intervention^*^	Post-intervention^*^	Z	P-value^†^
	Median (Range)	Median (Range)		
Total cohort (N = 256)	77.2 (19.3 – 100.0)	96.5 (57.9 – 100.0)	12.599	<0.001
Gender				
Male (n=119)	77.2 (22.8 – 100.0)	94.7 (57.9–100.0)	8.186	<0.001
Female (n=137)	77.2 (19.3 – 98.3)	96.5 (63.2–100.0)	9.582	<0.001
Highest educational level				
High school (n= 14)	75.4 (22.8 – 98.3)	90.4 (61.4–100.0)	2.074	0.038
Diploma (n= 39)	77.2 (19.3 – 100.0)	96.5 (73.7–100.0)	4.913	<0.001
Bachelor's degree (n= 128)	77.2 (29.8 – 100.0)	94.7 (59.7–100.0)	9.137	<0.001
Master's degree and above (n= 75)	77.2 (38.6 – 98.3)	96.5 (57.9–100.0)	6.751	<0.001
Professional role				
Nurse (n= 117)	75.4 (19.3 – 98.3)	93.0 (59.7–100.0)	8.606	<0.001
Physician (n= 77)	75.4 (38.6 – 98.3)	96.5 (57.9–100.0)	6.912	<0.001
Allied health professional (n= 62)	80.7 (22.8 – 100.0)	98.2 (71.9–100.0)	6.142	<0.001
Length of PHC experience (years)				
< 3 (n= 25)	70.2 (22.8 – 100.0)	89.5 (61.4–100.0)	3.872	<0.001
3 – 10 (n= 120)	77.2 (19.3 – 98.3)	94.7 (57.9–100.0)	9.044	<0.001
11 – 20 (n= 90)	81.6 (36.8 – 100.0)	98.2 (64.9–100.0)	7.58	<0.001
> 20 (n= 21)	73.7 (54.4 – 96.5)	91.2 (61.4–100.0)	2.441	0.015
Previous training in patient experience improvement				
Yes (n= 102)	82.5 (36.8 – 100.0)	82.5 (36.8–100.0)	7.095	<0.001
No (n=154)	73.7 (19.3 – 98.3)	73.7 (19.3–98.3)	10.279	<0.001

**Figure 2:**
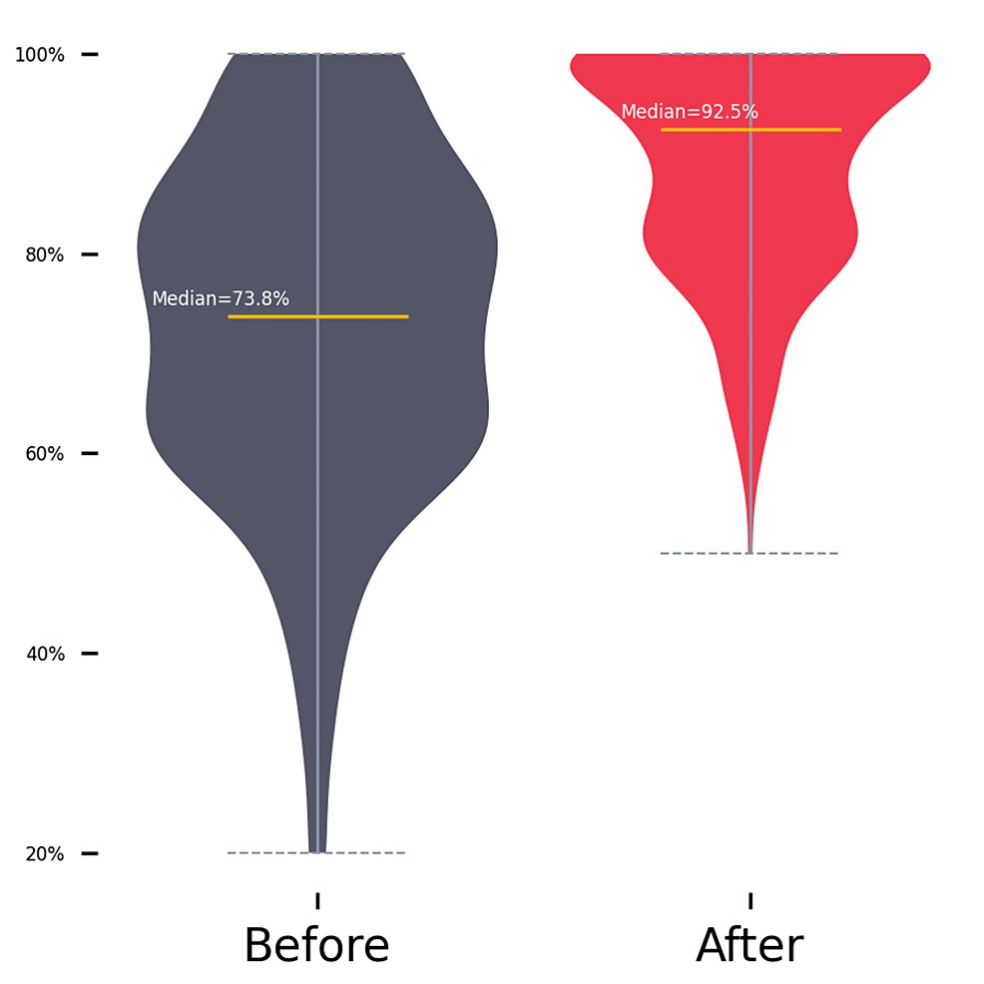
Distribution of pre-intervention (before) and post-intervention (after) attitude scores.

Moreover, practices and attitudes toward patient experience scores also demonstrated a statistically significant improvement (Z = 11.495, p < 0.001) with a large effect size (r = .72). The median post-intervention scores (median = 92.5) were notably higher than the median pre-intervention scores (median =73.8). Figure 2 shows the distribution of pre-intervention and post-intervention attitude scores using a violin plot. Table 3 displays the subgroup analysis for the attitude scores.

**Table 3:** Subgroup analysis for attitude scores. PHC: Primary health care; ^*^: percentages; ^†^: Wilcoxon signed-rank test

Characteristics	Before^*^	After^*^	Z	P-value^†^
	Median (Range)	Median (Range)		
Total cohort (N = 256)	73.8 (20.0 – 100.0)	92.50 (50.0 – 100.0)	11.495	<0.001
Gender				
Male (n=119)	72.5 (20.0 – 100.0)	92.5 (60.0 – 100.0)	8.02	<0.001
Female (n=137)	75.0 (20.0 – 100.0)	90.0 (50.0 – 100.0)	8.211	<0.001
Highest educational level				
High school (n= 14)	73.8 (50.0 – 97.5)	92.5 (65.0 – 100.0)	2.515	0.012
Diploma (n= 39)	70.0 (30.0 – 100.0)	90.0 (70.0 – 100.0)	4.935	<0.001
Bachelor's degree (n= 128)	75.0 (20.0 – 100.0)	92.5 (57.5 – 100.0)	7.812	<0.001
Master's degree and above (n= 75)	72.5 (32.5 – 100.0)	90.0 (50.0 – 100.0)	6.351	<0.001
Professional role				
Nurse (n= 117)	72.5 (20.0 – 100.0)	90.0 (50.0 – 100.0)	7.793	<0.001
Physician (n= 77)	75.0 (32.5 – 100.0)	90.0 (60.0 – 100.0)	6.434	<0.001
Allied health professional (n= 62)	77.5 (20.0 – 100.0)	95.0 (62.5 – 100.0)	5.514	<0.001
Length of PHC experience (years)				
< 3 (n= 25)	80.0 (47.5 – 100.0)	90.0 (62.5 – 100.0)	2.665	<0.001
3 – 10 (n= 120)	72.5 (30.0 – 100.0)	92.5 (50.0 – 100.0)	8.149	<0.001
11 – 20 (n= 90)	76.3 (20.0 – 100.0)	92.5 (65.0 – 100.0)	6.951	<0.001
> 20 (n= 21)	75.0 (32.5 – 100.0	90.0 (60.0 – 100.0)	3.225	<0.001
Previous training in patient experience improvement				
Yes (n= 102)	72.5 (20.0 – 100.0)	92.5 (60.0 – 100.0)	7.361	<0.001
No (n=154)	75.0 (20.0 – 100.0)	90.0 (50.0 – 100.0)	8.845	<0.001

**Figure 3:**
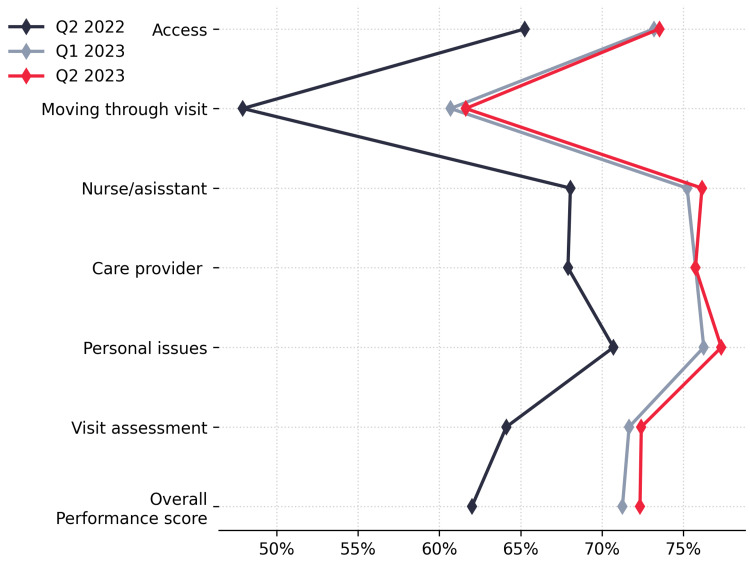
Comparing patient experience scores across various domains over three quarters.

Regarding patient experience, as captured by the patient experience system, Figure 3 displays positive trends in the system's assessment of facility performance across various domains. The overall performance score increased from 62% (Q2 2022) to 72.42% (Q2 2023).

**Figure 4:**
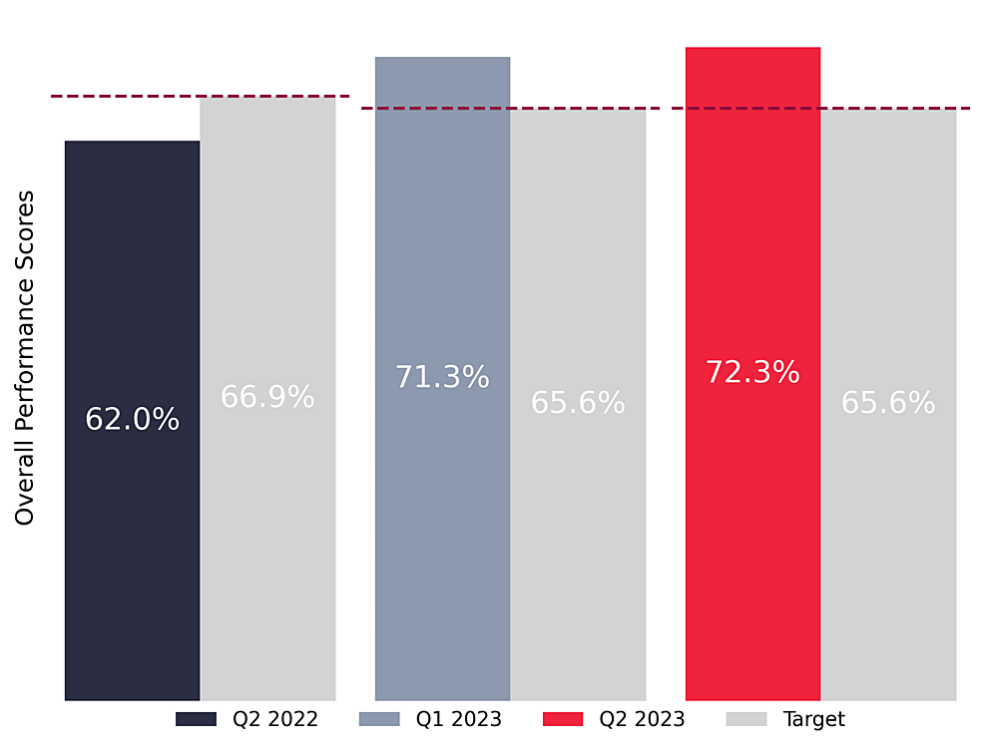
Overall patient experience performance score and target comparison over three quarters.

Notably, as shown in Figure 4, the overall performance score for Q2 2023 not only showed a significant improvement from the baseline but also surpassed the quarterly target.

Positive experiences with access to care rose from 65.26% (Q2 2022) to 73.53% (Q2 2023), while moving through the visit showed improvement, increasing from 47.92% (Q2 2022) to 61.62% (Q2 2023). Positive interactions with nursing and assistant staff increased from 68.06% (Q2 2022) to 76.15% (Q2 2023), and positive experiences with care providers rose from 67.91% (Q2 2022) to 75.75% (Q2 2023). Moreover, positive experiences in the domain of personal issues increased from 70.71% (Q2 2022) to 77.34% (Q2 2023). The overall assessment of the visit also improved, increasing from 64.12% to 72.42%.

**Figure 5:**
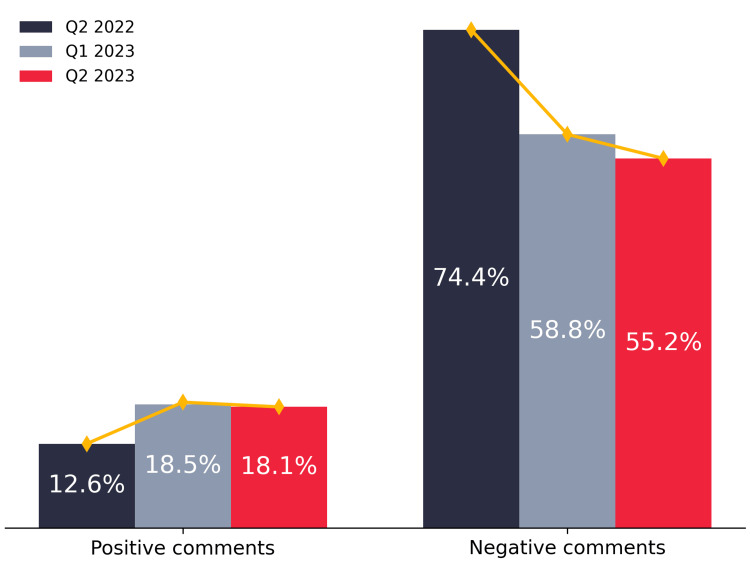
Comparison of positive and negative comments percentages over three quarters (excluding mixed and neutral comments).

In Figure 5, the comments metric indicates an increase in positive comments, rising from 12.6% of all comments received in Q2 2022 to 18.1% in Q2 2023. Concurrently, negative comments significantly decreased, dropping from 74.4% of all comments in Q2 2022 to 55.2% in Q2 2023.

## Discussion

Our study evaluated the impact of a comprehensive training program on frontline clinical staff's knowledge, perception, and attitude toward patient experience and service excellence. It also examined the effect of the training on patient experience metrics. The results showed significant improvements in knowledge, perception scores, and attitudes toward patient experience, indicating the intervention's effectiveness with a large effect size.

Frontline clinical staff play a significant role in shaping the patient experience, and our targeted training program aimed to equip them with essential skills for delivering patient-centered care. The increase in knowledge and perception scores indicates the program's effectiveness in enhancing staff understanding of patient experience aspects, which is important for providing quality care.

Additionally, the improvement in attitudes toward patient experience is a positive finding. Positive attitudes are important for fostering a patient-centered culture within healthcare settings, where patient comfort, satisfaction, and involvement in their care journey are prioritized. The positive changes in attitudes following the intervention suggest that the program successfully promoted a patient-centric approach among the frontline clinical staff.

The patient experience system's assessment showed positive trends and significant improvements across all domains. The overall performance score notably increased from the baseline and surpassed the quarterly target, indicating the intervention's effectiveness in enhancing patient experience. The increase in positive experiences with access to care, interactions with staff, and care providers highlights the program's positive impact on patient-staff interactions, along with improved experiences related to personal issues, addressing individual patient needs and preferences.

Analysis of comments from the patient experience system showed a significant increase in positive comments and a decrease in negative comments. This aligns with the improved patient experience scores, validating the training program's success in enhancing the overall patient experience.

Healthcare quality is a complex concept often misunderstood as vague terms like "goodness" or "luxury," making it challenging for patients to precisely define it [19]. Patient experience, on the other hand, is a multifaceted aspect of healthcare quality, reflecting feedback from patients on their actual experiences of care and treatment, combining objective facts with subjective views. Patients assess service quality by comparing their pre-service expectations with their actual experience, known as the "gap," which influences their perception of service quality [5,14]. Patient perception of service quality, i.e., patient experience, can be heavily influenced by factors such as waiting times, access to care, personalized attention, and the quality of interactions with clinical and non-clinical staff. These elements can significantly impact overall patient satisfaction and perception of care quality, often outweighing the importance of clinical services provided [1,5].

Our study contributes to the growing body of literature on patient experience and healthcare quality improvement (QI), specifically focusing on training frontline healthcare providers for QI and enhancing patient experience and outcomes. Our findings align with a systematic review of 39 studies on teaching QI to clinicians, which indicated clear improvements in participants' knowledge and confidence in performing QI. However, the review found limited evidence of the impact of educational outcomes on the clinical effectiveness of the interventions [20].

In addition, results from an experiential training program in QI for Practicing Professionals, conducted over 10 years, revealed positive changes in clinical practice and benefits to patients. The program was well-received by trainees, who valued the acquired skills [21].

As mentioned in the Introduction, the literature has presented mixed findings regarding the impact of QI strategies on clinical outcomes [6-13]. For instance, a recent Cochrane review comprising 553 trials investigating the effects of QI strategies on outcomes of adults with diabetes revealed that provider-targeted QI strategies, such as clinician education, demonstrated modest effects when implemented alone. However, when combined with other strategies, these provider-targeted QI approaches led to meaningful clinical improvements [22].

Our study emphasizes the significance of patient experience as a crucial outcome in healthcare. The positive results highlight the potential of targeted training programs for healthcare professionals to enhance patient experience and create a patient-centric care environment. Prioritizing patient satisfaction, comfort, and involvement in their care journey is essential. Timely access to care, respect for privacy, and personalized treatment plans are vital components of exceptional patient experience. Moreover, effective communication, empathy, and patient education can greatly enhance therapeutic relationship and overall satisfaction. Achieving first-class healthcare requires a delicate balance between clinical excellence and patient-centered care. By embracing both aspects, healthcare systems can ensure better medical outcomes and a more fulfilling experience for patients.

Limitations

The present study has several limitations. First, the lack of a control group limits our ability to definitively attribute the observed improvements in patient experience solely to the intervention. While the positive outcomes suggest the effectiveness of the targeted training program, future studies with a control group could provide more robust evidence by isolating the intervention's effects from other potential influences.

Second, the Hawthorne effect, wherein participants may alter their behavior due to being aware of being observed, cannot be ruled out as a possible influence on the results. Despite efforts to minimize such effects, the awareness of participating in a study focused on patient experience improvement may have influenced the attitudes and behaviors of the frontline clinical staff during the assessment period.

Additionally, our study was conducted in a specific setting, which may limit the generalizability of the findings to other healthcare contexts. The reliance on unsupervised self-reports for data collection introduces the possibility of response bias and individual variations in reporting.

## Conclusions

In conclusion, our study demonstrated the positive impact of a comprehensive training and education program on frontline clinical staff's knowledge, perception, and attitude toward patient experience, and subsequent improvements in patient experience scores. The significant increase in positive patient feedback across various domains underscores the importance of patient-centered care in achieving clinical excellence and enhancing overall healthcare quality. These findings suggest that focused interventions could potentially enhance patient experience and support quality improvement efforts in healthcare settings.
